# A Comparative Evaluation of Microleakage of Glass Ionomer Cement and Chitosan-modified Glass Ionomer Cement: An *in vitro *Study

**DOI:** 10.5005/jp-journals-10005-1225

**Published:** 2014-04-26

**Authors:** Deena Abraham, Abi Mathew Thomas, Saroj Chopra, Stephen Koshy

**Affiliations:** Senior Resident, Department of Pedodontics and Preventive Dentistry Christian Dental College, Ludhiana, Punjab, India; Professor and Head, Department of Pedodontics and Preventive Dentistry Christian Dental College, Ludhiana, Punjab, India; Professor, Department of Pedodontics and Preventive Dentistry Christian Dental College, Ludhiana, Punjab, India; Associate Professor, Department of Orthodontics and Dentofacial Orthopedics Christian Dental College, Ludhiana, Punjab, India

**Keywords:** Microleakage, Glass ionomer cement, Chitosan modified GIC, Thermocycling

## Abstract

**Objective: **To do a comparative study of microleakage of glass ionomer cement (GIC) and chitosan modified glass ionomer cement and evaluate which exhibited lesser microleakage.

**Materials and methods: **Sixty freshly extracted sound primary molar teeth were obtained. Two groups of samples were created for the study which comprised of group I (glass ionomer cement—GIC) and group II (Chitosan modified glass ionomer cement). Class V cavities were prepared on the buccal surfaces. All the tooth surfaces except the restoration and a 1 mm zone adjacent to its margins were covered with two coats of varnish. The specimens were then immersed in 2% basic fuschin dye solution for 24 hours. The teeth were sectioned into two halves buccolingually in an occlusoapical direction. Sections were viewed under stereomicroscope and the degree of microleakage was evaluated using specific scoring criteria. For comparative evaluation of microleakage scores between glass ionomer cement and chitosan modified cement, a nonparametric Mann-Whitney statistical analysis was done.

**Results: **Statistical analysis showed no significant differences between groups I and II with the p-value at >0.05.

**Conclusion: **Chitosan modified GIC holds great promise for general dentistry as a future restorative material with microleakage properties similar to or better than GIC.

**How to cite this article: **Abraham D, Thomas AM, Chopra S, Koshy S. A Comparative Evaluation of Microleakage of Glass Ionomer Cement and Chitosan-modified Glass Ionomer Cement: An *in vitro *Study. Int J Clin Pediatr Dent 2014;7(1):6-10.

## INTRODUCTION

Dental caries is an age old disease which has been the bane of affiction in the oral cavity. Due to lack of oral health awareness and frequent ingestion of refined carbohydrates, caries is most commonly seen affecting pediatric patients. Restoring the carious lesion at an early stage is an ideal treatment option in order to preserve the primary teeth until its normal anticipated exfoliation. This helps to assist in the maintenance of a healthy oral environment and arch length as well as to preserve the function of mastication and speech.^1^

However, even a simple restorative treatment plan is likely to evoke anxiety in a pediatric patient and may prove to be a challenge to the clinician. Hence when choice of the restorative material is made, simplicity of clinical appli­cation of the material should be considered along with other properties of the restorative material. The interest in the clinical use of glass ionomer cements (GIC) arose mainly from their particular advantage of requirement of a short time to fll the cavity which is a desirable property while treating young children.^2^

Condensable or high-viscosity glass ionomer cements, developed early in the 1990s, as filling materials in the atraumatic restorative therapy technique, were desirable due to their advantageous properties like faster setting, adequate strength and polishability in a single visit. However, the risk of fracture exists for large restorations.^3^ High-viscosity glass ionomers are still inferior to resin-based restorative materials when it comes to fracture toughness.^4^ Hence, there has been a constant quest for further improvement in the properties of the material while retaining its multitude of clinical advantages.

It was reported in 2007 that the flexural strength of a commercial GIC was significantly improved by the addition of chitosan. Moreover, in the presence of chitosan, it was found that release of fluoride ions from GIC was catalyzed.^5^Chitosan is a partially or completely deacetylated derivative of chitin. Researchers have demonstrated its great potential for a wide range of uses due to its versatile chemical and physical properties like biodegradability, biocompatibility, antimicrobial activity, nontoxicity.^6,7^

However, addition of any agent into a material in an attempt to improve the properties, should not jecopardize any other desirable property of the parent material. An important property responsible for the success of a material used for restorative purposes in the oral cavity is its ability to bond to tooth structure in a way that there is a complete and perfect seal between the margins of restorations and tissue of the tooth. A measure of this property is microleakage. Glass ionomer cement has chemical bonding to tooth structure. Hence a good adhesion to tooth lowers the risk of microleakage at the margins. The addition of chitosan in an attempt to improve the physical properties should not interfere with the adhesive property and escalate the risk of microleakage at the margins of the restoration.

The objective of this study was to evaluate the micro-leakage of chitosan modified glass ionomer cement and compare it to glass ionomer cement and thereby come to a conclusion if it performed at par or better than unmodified glass ionomer cement with regard to microleakage.

## MATERIALS AND METHODS

The materials used in the study were self curing glass iono-mer cement (Fuji IX, GC, Tokyo, Japan). The experimental cement was formulated from the same batch; by incorporation of 10% v/v Chitosan (HiMedia Laboratories, Mumbai, India) into liquid component of glass ionomer cement, after dis­solving it in 1% acetic acid.

### Collection of Samples

For the assessment of microleakage a total of sixty sound primary molar teeth were obtained. The teeth used for the study were obtained from patients having retained molars indicated for extraction, after taking parental consent for the treatment. The extracted teeth were cleaned of soft tissue and debris and stored in saline at room temperature.^8^ The teeth were disinfected by immersion in 1% Chloramine T solution (SD Fine-Chem Limited, Mumbai, India) for 1 week and then washed and dried.^9^

The samples were divided into the following groups:

- Group I: Glass ionomer cement (Fuji IX).
- Group II: Chitosan modified glass ionomer cement.

### Preparation of Class V Cavities

Class V cavities 4 mm wide × 2 mm high × 1.5 mm deep^10^were prepared on the buccal surfaces of teeth with no reten­tive features incorporated in the cavity design, using burs (No. 1 round bur, No. 57 straight fissure bur) with high speed air rotor handpiece with water coolant. All cavosurface mar­gins were kept at 90° without bevel designs and burs were changed after every five preparations.^9,11^ The standardiza­tion of cavities was done using a divider, dial callipers, and a graduated probe to further confirm the depth of cavity.

### Restorations of the Cavities in Groups I and II

The prepared cavities were restored in both groups with the respective materials after conditioning. GC dentin condi­tioner was applied for 20 seconds to the cavity walls using a brush with light scrubbing motion, followed by rinsing and drying by directing the air stream from the sides to avoid the desiccation of dentin. The cavities were restored and varnish was applied.

### Preparation for Assessment of Microleakage

After restoration, the teeth was stored in distilled water at 37°C for 24 hours^12,13^ and then subjected to 1500 thermo-cycles 11 to 14 at 5°C and 60°C, with 20 seconds of dwell time in each bath.^11^

Following thermocycling the specimens were prepared for immersion in dye solution. All the tooth surfaces except the restoration and a 1 mm zone adjacent to its margins were covered with two coats of varnish. The root apices if any, were sealed with sticky wax.^13^ The coated teeth were then immersed in 2% basic fuchsin dye solution (Ranbaxy Fine Chemicals Ltd, India) for a period of 24 hours at 37°C. After removal from the dye, the coatings were stripped from the teeth by peeling and where necessary, by scraping. The teeth were then thoroughly washed in water, dried and then were mounted in acrylic resin prior to sectioning.

The teeth were sectioned into two halves buccolingually in an occlusoapical direction through the middle of resto­ration by using a diamond disk mounted on a straight hand piece with water coolant. Each section was then observed under a stereomicroscope (Leica M 80) with a magnification of 30×. The degree of microleakage of both halves was assessed. The section showing the maximum degree of dye penetration was chosen for grading the microleakage. Scoring of each speci­men along the tooth restoration interface was recorded by two evaluators. If disagreement occurred between the evaluators, a consensus was obtained after re-examination of the specimen by both investigators.

The extent of the microleakage was noted according to the following scoring criteria^9^:

1. No marginal leakage
2. Up to 1/3 cavity depth
3. 1/3-2/3 cavity depth
4. >2/3 cavity depth but not involving the axial wall.
5. Involving the axial wall.

### Statistical Analysis

All data was statistically analyzed (SPSS 15.0, IBM) using a nonparametric Mann-Whitney test. Statistical significance was taken as p < 0.05.

**Fig. 1:**
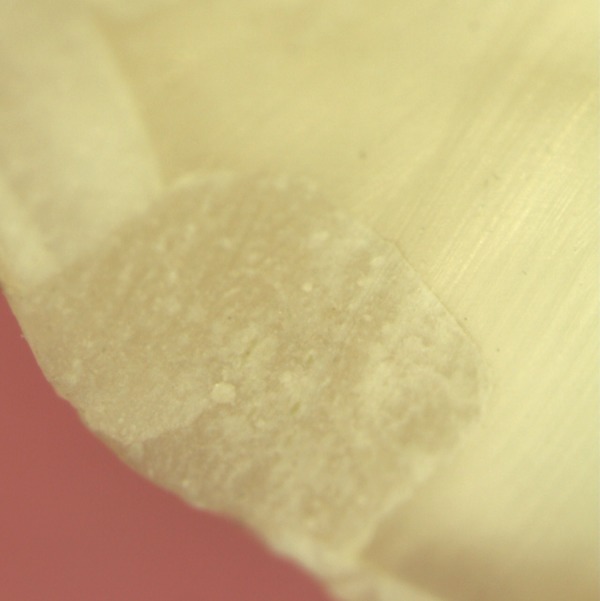
Score 0: No dye penetration

**Fig. 2:**
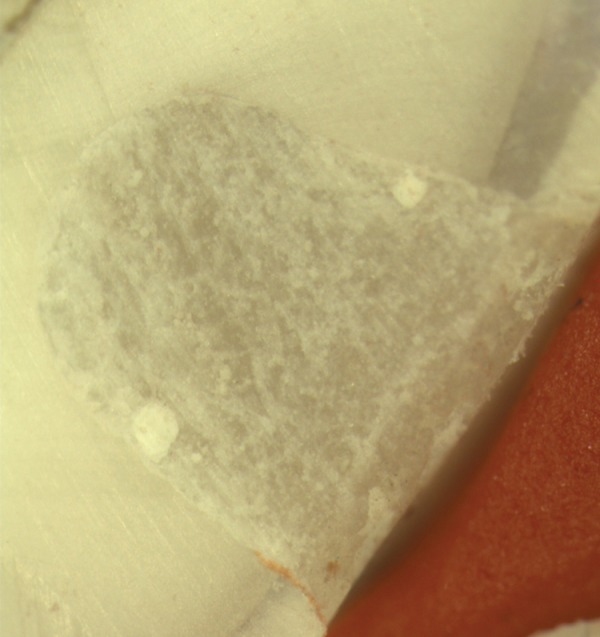
Score 1: Up to 1/3rd dye penetration depth

**Fig. 3:**
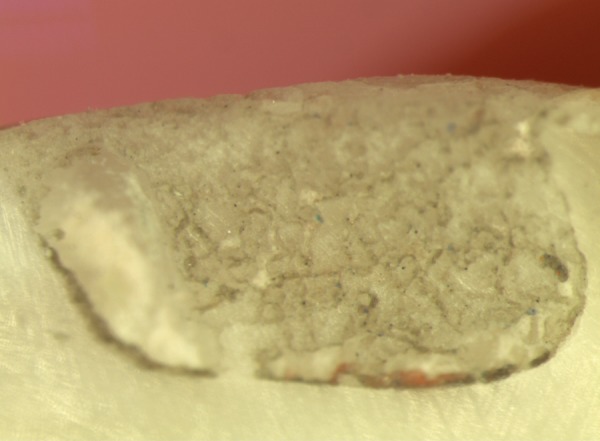
Score 4: Dye penetration involving the axial wall

**Graph 1:**
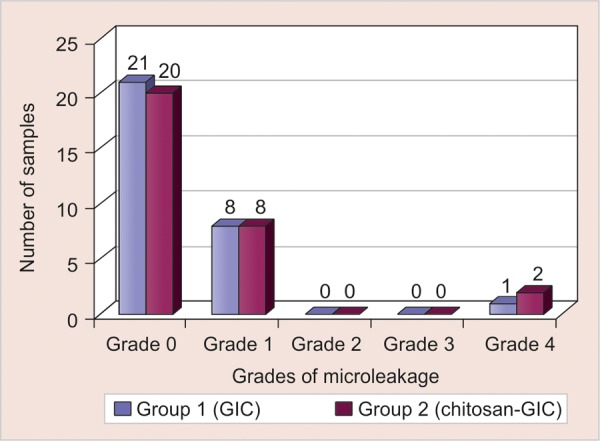
Comparison of microleakage observed with glass ionomer cement and chitosan modified glass ionomer cement

## RESULTS

A majority of the samples in groups I and II did not reveal any microleakage with a score of 0 as depicted in Figure 1. However eight samples in each group revealed compara­tively minimal degrees of microleakage of a score of 1 as seen in Figure 2. And one sample of group I, compared to two samples in group II revealed a high microleakage status of score 4 (Fig. 3).

Of the 30 samples investigated in either group, none exhibited dye penetration between 1/3rd and 2/3rd of the cavity depth (score 2 ) or greater than 2/3rd of cavity depth, but not involving the axial wall (score 3) as noted in Table 1 and Graph 1. Based on the scores that were tabulated, the p-value was >0.05 (see Table 1), thus indicative that there was no significant difference between microleakage of glass ionomer cement and chitosan modified glass ionomer cement.

**Table 1:** **Table 1: **Comparison of microleakage observed with glass ionomer cement (group I) and chitosan modified glass ionomer cement (group II)

*Group*		*Grade of microleakage*										*Total (n)*		*p-value*	
		*0*		*1*		*2*		*3*		*4*					
Group I		21		8		–		–		1		30					
Group II		20		8		–		–		2		30		0.730	

p = 0.730; not significant

## DISCUSSION

The results of this study reveal that incorporation of 10% (v/v) of chitosan in glass ionomer cement did not lead to a significant increase in the microleakage of the material. There was no statistically significant difference in the mean microleakage values between groups I and II. This could have transpired because the minor amount of chitosan did not hinder the formation of bond of glass ionomer with the tooth structure.

These results were in harmony with the fact that Fuji IX has a coefficient of thermal expansion close to that of tooth. Only one sample showed dye penetration involving axial wall which could be due to the failure of adhesion between GC Fuji IX and tooth because of incorporation of void/ air bubble during the bulk placement of material into the cavity.

Frankenberger et al^3^ reported that Fuji IX sets faster and is of higher viscosity because of finer glass parti­cles, anhydrous polyacrylic acids of higher molecular weight and a high powder to liquid ratio. These properties may be responsible for Fuji IX exhibiting a good marginal seal.

In 2007, Petri et al^5^ observed the improved mechanical properties of GIC, on adding small amounts of chitosan into GIC. It was suggested that if the interfacial tension between each component is high, or in other words, the adhesion between each component is weak, the mechanical properties are poor. Therefore, an additive like chitosan would lead to formation of networks with polyacrylic acid around the inorganic particles which reduce the interfacial tension among the GIC components, thus improving mechanical performance. This effect was explained based on a model where a polymeric network binds strongly around the inorganic filler.

In 2012, Elsaka^14^ reported a study evaluating the anti­microbial activity and the adhesive property of dental adhesive containing various incremental concentrations of chitosan. It was reported that these properties improved upon addition of small amounts of chitosan. Upon addition of 0.12 and 0.25% (w/w) chitosan , the microtensile bond strength values were better compared to the control group, however there were no significant differences.

Berger et al^15^ proposed that networks containing covalently crosslinked chitosan could be considered as smart hydrogels undergoing a reversible discontinuous volume phase change in response to external physicochemical factors like temperature and pH. This would in turn negate any microleakage tendency of the cement.

Since *in vitro *studies do not reflect all variables present in the mouth, the results of the present *in vitro *study cannot be extrapolated to the clinical situation unless adequate clinical trials are conducted to test the *in vivo *Efficacy of the material. Further tests should be undertaken to compare and evaluate the other strength characteristics like flexural strength, bond strength and properties like hardness, setting and working times of the experimental cement. Studies should also be carried out to evaluate the shelf life and stability of chitosan modified glass ionomer cement.

Based on the reported benefits of improved strength and fluoride release, along with conclusions drawn from the present *in vitro *study of favorable microleakage results; chitosan modified glass ionomer cement can be considered as a promising restorative material. It holds a place in the application of posterior occlusal restorations, in minimal invasive techniques as well as for general clinical utility in pediatric dentistry.

## CONCLUSION

1. The experimental cement containing 10% (v/v) of chitosan exhibited microleakage comparable to the unmodified cement.
2. Incorporation of 10% (v/v) of chitosan had no delet­erious effect on the microleakage of glass ionomer cement. Hence chitosan modified glass ionomer cement stands out as a promising restorative material for future applications.
